# Oesophageal Perforation: A diagnostic and therapeutic challenge in a resource limited setting. A report of three cases

**DOI:** 10.1186/1749-8090-6-116

**Published:** 2011-09-25

**Authors:** Deo D Balumuka, Phillipo L Chalya, William Mahalu

**Affiliations:** 1 Department of Cardiothoracic Surgery, Weill-Bugando university college of Health sciences, P.O.Box 1464, Mwanza, Tanzania; 2 Department of Surgery, Weill-Bugando University College of Health Sciences, P. O.Box, 1464, Mwanza, Tanzania

## Abstract

**Background:**

Oesophageal perforation is a condition associated with a high mortality. Its management is still controversial with operative treatment being favoured but a shift to conservative management is occurring. Very little exists in medical literature about its management in Sub-Saharan Africa, where the paucity of thoracic surgeons is compounded by limited diagnostic and therapeutic facilities.

**Case Presentation:**

We report three cases of oesophageal perforation which were all treated conservatively with tube thoracostomy, nil by mouth with feeding gastrostomy, intravenous antibiotics and chest physiotherapy. Two patients achieved oesophageal healing but one died due to severe septicaemia.

**Conclusion:**

In a resource restricted setting, conservative management which includes enteral nutrition by feeding gastrostomy, tube thoracostomy to drain inter pleural contaminants, intravenous antibiotics and chest physiotherapy is a safe and effective treatment for oesophageal perforations.

## Background

Oesophageal perforation is an uncommon but potentially fatal injury that can quickly progress to mediastinitis, sepsis and multiorgan failure, if early recognition and proper treatment is not instituted [1]. The commonest cause of oesophageal perforation is instrumentation. The frequent use of upper gastrointestinal fiberoptic endoscopy has led to an increase in the actual number of perforations [2]. The most common area of perforation is in the region of the cricopharyngeus muscle. The oesophageal inlet is the narrowest area of the oesophagus and the cricopharyngeus muscle contributes to the decrease in diameter of the lumen [2]. The next commonest site is the lower oesophagus as it narrows to pass through the hiatus[3]. Dilations of the oesophagus carry a risk of perforation, because most are performed for stricture and perforations occur in the diseased thoracic or abdominal portion of the oesophagus [2]. Sever perforations can be caused by; attempted foreign body removal either by a poorly trained endoscopist, or by one who tries to push the foreign body ahead of the endoscope in to the stomach too vigorously[2] or by using the wrong equipment in a resource restricted area.

However, most literature comes from western institutions with little coming from Sub-Saharan Africa. We present three cases of oesophageal perforations in a limited diagnostic and therapeutic facility with the aim of showing the feasibility of conservative management in a resource restricted setting.

## Case 1

A male African of 11 years who had ingested corrosive material 7 years ago, used by the mother to treat her hair, presented for a repeat dilation of a lower oesophageal stricture. Eight hours following the dilation he developed chest pain and fever. These complaints had lasted for three hours and were increasing. Examination revealed a temperature of 39.8 degrees centigrade and respiratory rate of 30 breaths per minute. Tracheal deviation to the right, normal vesicular breath sounds in the right hemi thorax. A stony dull percussion note on the left hemi thorax and no air entry were detected. Blood pressure was normal as was the rest of the exam. Urgent chest radiograph showed a left hydropneumothorax, pneumomediastinum with a normal right hemi thorax. Barium oesophagram could not be done because the mother could not afford it.

A diagnosis of acute mediastinitis due to perforated lower oesophagus was made. Left tube thoracostomy was inserted and it drained the fruit juice the child had drunk. Intravenous Metronidazole and Ceftriaxone were prescribed. Nil by mouth was advised and Intravenous fluids were prescribed. Septicaemia persisted for 6 days, following which a feeding gastrostomy tube was inserted, and enteral feeding was started through it. Chest physiotherapy was instituted to facilitate drainage of thoracic contaminants.

After two weeks we attempted feeding orally but this resulted in a fever, cough and increased thoracostomy drainage and chest radiograph showed features of left lower lobe pneumonia. He was started on another course of broad spectrum intravenous antibiotics (gentamycin and ampicillin), chest physiotherapy and with in a week he had recovered. At four weeks (1 month) from the day of presentation, he was made to swallow 10 mls of methylene blue to check for healing of the oesphagus. Barium oesophagram could not be done, because the mother a widow, could not afford it.

Following the methylene blue swallow the patient was observed for 2 days and there was no leakage into the thoracostomy tube, fever or cough. At this time (1 month) after the perforation oesophageal healing was confirmed. Oral feeding was instituted and after another two days of observation, the chest tube and feeding gastrostomy tube were removed. He was discharged and sent home feeding orally without any complications at all. Three months following discharge he reported to the clinic with a gastrostomy stitch sinus. This was managed surgically and he was discharged feeding orally. The family was not financially able to afford an oesophagram as recommended to assess the state of the oesophagus at discharge; the mother also believed it was not needed if the child was feeding properly.

## Case 2

A 7 year old female with a history of having swallowed a coin was referred from a district hospital where they had attempted to remove the coin with forceps and failed. The radiograph from the district hospital showed the coin to be behind the clavicles. At our centre, rigid oesophagoscopy was done and the coin was removed minus complications. An area of hyperaemia was noted about 1 cm in diameter just above the coin. The patient was sent to the ward for observation. On the ward she had some chest pain following feeding, but nothing else was noted and the next day the parents asked for her to be discharged, which was granted. Once home the patient developed a high grade fever, dyspnoea, cough and worsening chest pain. A chest radiograph done at the district hospital, showed a right hydropneumothorax. The left hemi thorax was normal. Aspiration of the contents of the right pleural cavity consisted of whitish contents. A diagnosis of tuberculosis with a tracheoesophageal fistula was made and treatment with anti-tuberculosis medication was started. Following failure of improvement after 18 days she was referred to our centre. She presented on day 18 from the incident with a high grade fever, chest pain and cough. The symptoms were worsened by feeding.

On arrival, examination revealed, fever 40 degrees centigrade, wasting and pallor. A respiratory rate of 31 breaths per minute and no tracheal deviation. Stony dull percussion and no air entry in right hemi thorax, a right thoracostomy tube which was actually an improvisation constructed by using a nasogastric tube connected to an effluent bag, contents consisted of food material. Blood pressure was 96/65 mmhg pulse of 119 bpm in sinus rhythm. Normal abdominal exam was noted. The chest radiograph showed pneumomediastinum and right hydropneumothorax. A diagnosis of cervical oesophageal perforation was made. Intravenous broad spectrum antibiotics were prescribed (Ceftriaxone and Metronidazole), a proper tube thoracostomy was inserted, nil by mouth was advised and intravenous fluids were given. A full blood picture, blood smear for malaria parasites and repeat chest radiograph were requested. Barium swallow could not be done because the machine had broken down. Her initial haemoglobin was 7 g/l and she was transfused 450 millilitres of whole blood. ESR was 30 mm/hr, she had no malaria parasites. The repeat radiograph showed lung expansion, even though there was still some contamination of the right hemi thorax. After five days of treatment the signs of septicaemia subsided and a feeding gastrostomy tube was inserted. Feeding was started by the enteral route, chest physiotherapy was continued and ambulation was encouraged. After two weeks, a barium oesophagram was done and showed no leak, oesophageal healing was confirmed. Oral feeding was started one day after this and both the chest tube and feeding gastrostomy tube were removed. With in two days, she was feeding well orally and she was discharged. The patient reported back to the clinic at 1 month with no further problems.

## Case 3

A 23 year old African male prisoner reported to our centre with a history of having forcefully pushed a piece of wood in his throat in an attempt to take his own life. He had initially been treated in the prison hospital, where antibiotics were given and oral feeding encouraged. After 15 days he was brought to our centre. On arrival, he complained of chest pain, cough with purulent foul smelling sputum, dysphagia, and odynophagia since the incident which was 15 days ago. There was an associated swinging fever, malaise and anorexia. On examination, he was wasted very ill looking and drooling foul smelling saliva from the oral cavity, pale and febrile at 38.5 degrees centigrade. Trachea was central. Respiratory rate of 24 breaths per minute, but right side was not moving with respiration. The left side had a normal examination; the right side had features of an effusion with no air entry. He had normal cardiovascular exam, save for a tachycardia of 112 bpm in sinus rhythm. The abdominal exam was also normal. The chest radiographs showed a right hydropneumothorax, no pneumomediastinum was noted, the left hemi thorax was normal. The barium oesophagram could not be done because the machine had broken down. A diagnosis of perforated oesophagus with a foreign body in situ was made. A tube thoracostomy was inserted and drained purulent material that was foul smelling. Nil by mouth with Intravenous fluids was prescribed. Broad spectrum intravenous antibiotics were prescribed for a period of 1 week. Blood for a complete blood picture was taken and showed low haemoglobin 9 g/l, leucocytosis with neutrophilia, other parameters were normal. The repeat chest radiograph did not show the piece of wood in the oesophagus, but showed a decrease in the right chest hydrothorax and some lung expansion. The patient was scheduled for oesophagoscopy in two days when the fever had subsided. This would also attempt to remove the foreign body. The oesophagoscopy revealed that the proximal part of the foreign body was at 15 cm from the incisors. The surrounding oesophagus was necrotic and friable. Removal failed because the foreign body was firmly attached and there was fear of tearing through vital mediastinal structures if excess force was used. A laparotomy was done for feeding gastrostomy tube insertion, but at opening the stomach, the distal end of the piece of wood was seen. It was gently pulled down and safely removed. It was 18 cm long and the widest part was 3 cm with the narrowest 0.5 cm end in the stomach. A feeding gastrostomy was there fore created.

On the ward he was advised to feed by gastrostomy and not orally, so as to reduce contamination and rest the oesophagus. Post laparotomy he continued to feed orally and by gastrostomy against the recommendations. This happened for 2 days and the patient started to deteriorate, having a high grade fever, productive cough and dyspnoea and died of sepsis three days later.

## Discussion

Oesophageal perforation is an uncommon but very dangerous injury mostly caused by instrumentation [1]. Treatment of oesophageal perforation depends on; the aetiology, site and size of perforation, the time from perforation to diagnosis, underlying oesophageal disease and the overall health of the patient. Prognosis is largely dependent upon the interval between perforation and treatment [3]. Those arriving late have worse outcomes compared to those who arrive and are treated early. As in our last case, the patient's overall health was not good physically nor mentally prior to arrival at our centre.

The decision to manage patients non-operatively (conservatively) or operatively (surgically) is largely controversial and the problem is compounded in the resource restricted areas in Sub Saharan Africa. Altorjay *et al *[4] suggested the following criteria for selection of non operative treatment.

1. Early diagnosis or leak contained if diagnosis delayed.

2. Leak contained within neck or mediastinum, or between the mediastinum and visceral lung pleura;

3. Drainage into oesophageal lumen as evidenced by contrast imaging;

4. Injury not in neoplastic tissue, not in abdomen, not proximal to obstruction;

5. Symptoms and signs of septicaemia absent and

6. Contrast imaging and experienced thoracic surgeon available

In the cases we managed none of the patients met the selection criteria stated above and yet two achieved oesophageal healing. This could be because children have a great propensity to heal [1]. But in a limited therapeutic facility, the only management that could be offered to such patients is conservative, especially in areas where thoracic surgeons are not available.

Nutritional support is of highest priority [1-10]. A nasogastric tube should not be used initially, as it may cause further injury at the site of perforation [3]. In our setting enteral nutrition, through a feeding gastrostomy is preferred, because it is easy to institute and very effective without the side effects of the expensive and unavailable intravenous nutrition. The insertion of the feeding gastrostomy tube in our setting was by the technique described by Stamm and usually needs the patient to be able to withstand the general anaesthesia, mostly halothane. The feeding tube used was actually an endotracheal tube. Size 7 for case 1, size 6.5 for case 2 and size 7.5 for case 3. We had to wait for the mediastinitis to subside, which took about 5 to 7 days. Antibiotics which are broad spectrum were prescribed for an average of 2 weeks (approximately 7-21 days). For every patient who had a fever, intravenous antibiotics were prescribed to contain the infection and also prevent worsening of the infection. The feeds given through the feeding tube consisted of millet porridge with mashed eggs, peanuts and milk, mashed plantain with mashed beans made sloppy by adding milk occasionally minced meat and mashed rice. Some times passion fruit juice and fresh milk with sugar was given between meals.

The patient who had an oesophageal stricture- case 1 had a long duration of antibiotics and a healing time of approximately 1 month, yet the patient with cervical oesophageal perforation case no 2 took 2 weeks to heal. The time of healing has ranged from 5 days to 3 months among patients with oesophageal perforations managed by the conservative method [1,3].

Prognosis is better in patients with normal oesophagus, prior to perforation compared to those with underlying oesophageal disease [1]. Cervical oesophageal perforations have a better outcome in general, showing a mortality rate of about 6% (0% to 10%). Thoracic and abdominal perforations were associated with higher mortalities 22% (0% to 44%) and 21% (0% to 43%) respectively. It is suggested that these differences in mortality rates is due to the containment of contamination by the fascial planes of the neck, following cervical perforations. By contrast, containment secondary to intra thoracic or intra abdominal oesophageal perforations is poor and results in early sepsis [3]. This was noted in two of our patients with thoracic perforations, who required a longer duration of antibiotics and time to heal- case 1 and also mortality due to severe sepsis in case 3. Perforations of more than 24 hours duration are associated with greater mediastinal contamination and hence more sepsis as seen in our 3^rd ^case.

Even though the interval from oesophageal perforation to initiation of treatment is a crucial determinant of prognosis, in a resource restricted setting, it is likely that more patients will present late i.e. over 24 hours (which predisposes them to more complications) and hence need for more aggressive management. One of the biggest problems faced by many medical practitioners in Sub Saharan Africa is the lack of proper therapeutic equipment which causes more harm unintentionally in these areas, as seen in our 2^nd ^case.

All the above cases could have been managed by operation as per the selection criteria by Altorjay et al, but in most Sub Saharan African centres there is a paucity of thoracic surgeons, hence the choice for conservative management, without the ability to convert to surgical management, even when deemed necessary.

Management of oesophageal perforations is based on retrospective series, mostly from the western world with little coming from Sub Saharan Africa. This fact may well be due to the rarity of the condition, such that only a few cases are encountered and managed.

But with the lack of therapeutic and diagnostic facilities, more patients will be presenting late and the only feasible mode of treatment may remain aggressive conservative management until there are more thoracic surgeons and better equipped centres. Referral to well equipped centres may be an option for some, however most patients cannot afford the transportation nor cost of treatment at these very few well equipped facilities. The limitation in the diagnosis and therapy in resource restricted centres may contribute to the poor outcome or even cause of perforations as in case no.2.

## Conclusion

In a setting of limited diagnostic and therapeutic facilities, with a paucity of thoracic surgeons, oesophageal perforations either early or late presenting should be considered for conservative management. Due to the unavailability of total parental nutrition and its complications, enteral nutrition via a feeding gastrostomy tube should be instituted. Broad spectrum intravenous antibiotics to control and prevent systemic sepsis should be given for as long as signs of infection persist. Tube thoracostomy should be placed to drain the inter pleural contamination. We believe the above choices are safe and feasible in a resource restricted setting for both adults and children.

## Consent

The authors confirm that written consent has been obtained from patient in order to publish the relevant clinical information included in the submitted manuscript.

## Competing interests

The authors declare that they have no competing interests.

## Authors' contributions

BDD is responsible for acquisition of data and writing the original manuscript. PLC and WM are responsible for conception and design as well as critical revision of the manuscript. All authors approved the final version submitted.

## References

[B1] Carissa L Carrie AL Adam JK Daniel JO Charles LS George WH Shawn D Oesophageal perforation in children: A review of one institutions experience Journal of Surgical Research 2010 164 13 17 10.1016/j.jss.2010.05.049 20850782

[B2] Luc M Hermes CG Ronald AM Oesophageal perforations Annals of thoracic surgery 1982 33 2 203 210 10.1016/S0003-4975(10)61912-1 7039537

[B3] Ghai A Wadher R Kamal K Verma V Mediastinitis after Oesophagoscopy: A Case report SAJAA 2009 15 2 33 34

[B4] Clayton J Brinster BA Sunil S Lawrence LM Blair M Larry RK John CK Evolving Options in the Management of oesophageal Perforation Annals of thoracic surgery 2004 77 1475 83 10.1016/j.athoracsur.2003.08.037 15063302

[B5] Verwoerd C Van-mazijk F Meyer JM A conservative approach in selected cases of late diagnosed oesophageal perforation Thorax 1977 32 232 234 10.1136/thx.32.2.232 867337 PMC470580

[B6] David B Skinner MD Alex G Little MD Tom R Demeester MD Management of Oesophageal Perforations The American journal of surgery 1980 139 760 764 10.1016/0002-9610(80)90379-7 7386730

[B7] Bradley L Bufkin MD Joseph I Miller MF Jr Kamal A Mansour MD Oesophageal perforations: Emphasis on Management Annals of thoracic surgery 1996 61 5 1447 51 10.1016/0003-4975(96)00053-7 8633957

[B8] Rosiere S Mulier A Khoury L Michael A Management of Oesophageal Perforation after Delayed Diagnosis: Merit of Tissue Flap Reinforcement Acta chir belg 2003 103 10.1080/00015458.2003.11679475 14653036

[B9] Stephen BV Robert R Tomas DM Patricia LA Oesophageal perforations in Adults. Aggressive, conservative Treatement lowers Morbidity and Mortality Annals of thoracic surgery 2005 241 6 1016 21 10.1097/01.sla.0000164183.91898.74 PMC1357179 15912051

[B10] Jeffrey L Michael S Robert JK Mathew B Nasser KA Thoracic Oesophageal Perforations: A decade of Experience Annals of thoracic surgery 2003 75 1071 4 10.1016/S0003-4975(02)04650-7 12683539

